# Boosting Lithium-Ion Storage Capability in CuO Nanosheets via Synergistic Engineering of Defects and Pores

**DOI:** 10.3389/fchem.2018.00428

**Published:** 2018-09-24

**Authors:** Zhao Deng, Zhiyuan Ma, Yanhui Li, Yu Li, Lihua Chen, Xiaoyu Yang, Hong-En Wang, Bao-Lian Su

**Affiliations:** ^1^State Key Laboratory of Advanced Technology for Materials Synthesis and Processing, Wuhan University of Technology, Wuhan, China; ^2^Laboratory of Inorganic Materials Chemistry, University of Namur, Namur, Belgium

**Keywords:** copper oxides, porous nanosheets, crystal engineering, anode, lithium ion batteries

## Abstract

CuO is a promising anode material for lithium-ion batteries due to its high theoretical capacity, low cost, and non-toxicity. However, its practical application has been plagued by low conductivity and poor cyclability. Herein, we report the facile synthesis of porous defective CuO nanosheets by a simple wet-chemical route paired with controlled annealing. The sample obtained after mild heat treatment (300°C) exhibits an improved crystallinity with low dislocation density and preserved porous structure, manifesting superior Li-ion storage capability with high capacity (~500 mAh/g at 0.2 C), excellent rate (175 mAh/g at 2 C), and cyclability (258 mAh/g after 500 cycles at 0.5 C). The enhanced electrochemical performance can be ascribed to the synergy of porous nanosheet morphology and improved crystallinity: (1) porous morphology endows the material a large contact interface for electrolyte impregnation, enriched active sites for Li-ion uptake/release, more room for accommodation of repeated volume variation during lithiation/de-lithiation. (2) the improved crystallinity with reduced edge dislocations can boost the electrical conduction, reducing polarization during charge/discharge. The proposed strategy based on synergic pore and defect engineering can pave the way for development of advanced metal oxides-based electrodes for (beyond) Li-ion batteries.

## Introduction

Lithium-ion batteries (LIBs) have been widely used to power various portable electronic devices since their commercialization (Masse et al., 2017). Now their performances are further improved for targeted use in (hybrid) electric vehicles and smart electric grids. Such emerging applications pose strict requirements on their energy/power density, cost, calendar life and safety, which can hardly be fully satisfied by current anode (mainly graphite) and cathode [LiCoO_2_ (Chai et al., 2017), LiMn_2_O_4_ (Xi et al., 2012), and LiFePO_4_ (Lu et al., 2011) etc.]. New anode and cathode materials have been testified to provide higher capacity and better cyclability as well as safety (Cai et al., 2016; Wu et al., 2016, 2017; Tan et al., 2017; Wang et al., 2017; Zhang Q. B. et al., 2018; Zheng et al., 2018). Meantime, new electrochemical energy storage devices, including sodium-ion batteries (Wu et al., 2016; Hu et al., 2017; Zhu et al., 2018), lithium-sulfur batteries (Li et al., 2018), and lithium-air batteries (Liu et al., 2016a,b; Wu et al., 2017) have also been proposed for large-scale applications in electric grid and storage of renewable energies. Among various new anode candidates for LIBs, copper oxide (CuO) has been considered to be promising because of its high theoretical capacity (670 mAh g^−1^), non-toxicity, low cost, and environmental friendliness (Zhang et al., 2014). However, the practical application of CuO has been plagued by its low conductivity and large volume change during continuous Li-ion insertion/extraction, giving rise to poor rate capacity and cycling stability.

To address these issues, various nanostructured CuO, such as nanoparticles (Hu et al., 2017), nanowires/nanorods/nanotubes (Yin D. M. et al., 2017; Yuan et al., 2017; Sun et al., 2018; Wang et al., 2018), porous and hollow nanostructures (Lyu et al., 2017; Peng et al., 2017; Tan et al., 2017; Yin H. et al., 2017; Zhang J. et al., 2018; Zhou et al., 2018), has been designed and synthesized to enlarge the electrode/electrolyte interface and shorten Li-ion diffusion paths. Particularly, porous ultrathin CuO nanosheets manifest combined merits of large exposed surface and anti-aggregation character of nanoparticles, enabling superior Li-ion storage capability. Additionally, coating or hybridization of CuO with conductive agents [e.g., carbon nanotubes (Cui et al., 2018), graphene (Chen et al., 2017; Li et al., 2017), amorphous carbon (Peng et al., 2017; Tan et al., 2017; Yin H. et al., 2017; Yuan et al., 2017] has been used to boost the electron transport within CuO-based composite electrode. However, the introduction of secondary additives may also bring some disadvantages. (1) It can complicate the whole synthetic procedure and thus increase processing cost. (2) The existence of non-uniform distribution of CuO and conductive component may impedance the mass transfer of electrolyte and Li-ion transport within the composite, leading to increased polarization. (3) The introduction of carbon may raise safety concerns due to the formation of Li-dendrites on carbon surface at high-rate charge/discharge and lower the volumetric energy density of the LIBs.

Besides surface conductive coating or compositing, crystal engineering is another promise way to boost the conductivity of CuO by manipulation of its crystallinity and crystallographic defects. For example, it has been reported that creating oxygen vacancy defects in TiO_2_ can significantly boost its sodium-ion storage property (Zhao et al., 2018). Generally, CuO synthesized by wet-chemical routes can possess relatively high surface area by avoiding undesired sintering at high temperatures. However, they may also contain a large number of structural disorder (e.g., dislocation), surface functional groups and foreign ligands, lowering electrical conduction. Rational engineering the defect content while retaining relatively high exposed surface can significantly promote the electron transport and Li-ion insertion/extraction, thus enabling enhanced electrochemical properties.

Inspired by these, in this paper we report the rational design and facile synthesis of porous ultrathin CuO nanosheets on a large scale by a mild solution process. We further engineer the crystallinity and defect contents in the resultant CuO product by a simple yet effective post-annealing process. Our results reveal the solution-derived CuO subject to mild annealing at 300°C exhibits an improved crystallinity with reduced dislocations while still maintains a high accessible surface due to the well-preserved porous nanosheet structure, which is beneficial for fast and reversible Li-ion storage even at high rates.

## Experimental

### Materials synthesis

All chemicals were analytically grade and used as received without further purifications. In a typical synthesis, 10.34 g Cu(NO_3_)_2_·3H_2_O was dissolved in 500 mL deionized water with magnetic stirring at room temperature. After stirring for 10 min, 3.45 g NaOH was added into Cu(NO_3_)_2_ solution. After reaction for 30 min, the resulting precipitate was heated at 80°C for 12 h. The resulting black-brown product was collected by filtration, washed with deionized water and absolute ethanol several times and finally dried at 120°C for 12 h in air.

The samples were further annealed at 300, 400, and 500°C for 1 h, respectively with a heating rate of 1°C /s. The corresponding products were denoted as CuO-300, CuO-400, and CuO-500, respectively for clarity.

### Characterization

X-ray diffraction (XRD) patterns were recorded on Bruker D8 diffractometer with a Cu Kα radiation (λ = 0.1542 nm). The morphology of the samples was observed using a Hitachi S-4800 scanning electron microscope (SEM). Transmission electron microscopy (TEM) and high-resolution TEM micrographs were operated on a JEM-2100F transmission electron microscope with an accelerating voltage of 200 kV. Brunauer-Emmett-Teller (BET) surface areas of the samples were derived using N_2_ adsorption tests on a Micromeritics ASAP 2020. Before adsorption, the samples were outgassed at 120°C overnight in a vacuum line. Pore size distribution was calculated from the desorption branch of the isotherms using the Barrett-Joyner-Halenda (BJH) method.

### Electrochemical measurements

The working electrodes were prepared by mixing active materials, carbon black (super-P) and PVDF binder with a weight ratio of 80:10:10 in NMP to form a slurry. The resultant slurry was uniformly coated on Cu foils and then dried at 70°C in vacuum for 12 h. 1 M solution of LiPF_6_ dissolved in ethylene carbon (EC)/dimethyl carbonate (DMC) (1: 1 w/w) was used as electrolyte. CR2025 coin cells were assembled with lithium foils as counter and reference electrodes in an argon-filled glovebox with water and O_2_ contents below 1 ppm. Galvanostatic charge-discharge (GCD) tests were recorded on a multichannel battery testing system (LAND CT2001A) within a potential range of 0.01~3.0 V (vs. Li^+^/Li). Cyclic voltammetry (CV) measurements were carried out using a CHI 760D electrochemical workstation at a scan rate of 0.2 mV/s. Electrochemical impedance spectra (EIS) tests were recorded on an Autolab workstation. Before EIS tests, the Li-half cells were activated at 0.2 C for 5 cycles (1°C = 670 mA/g). All electrochemical tests were carried out at room temperature.

## Results and discussion

**Figure 1:**
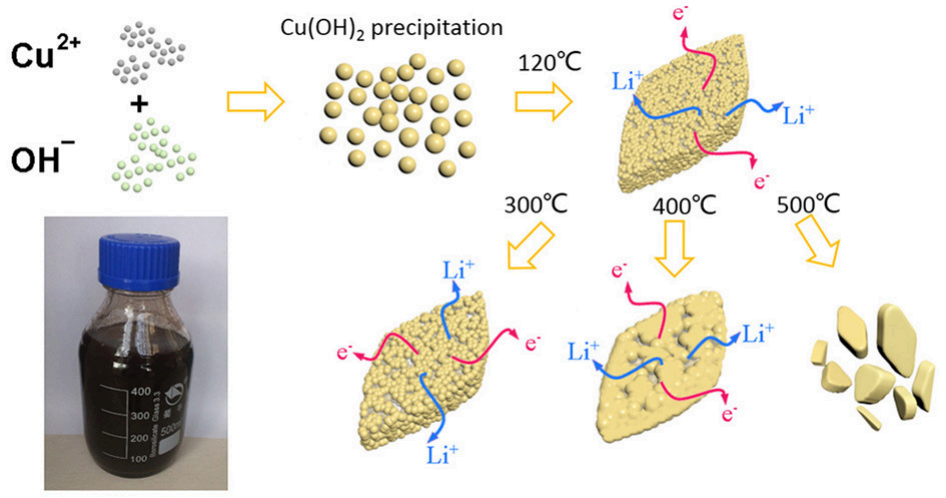
Schematic synthesis process of CuO nanosheets by a solution route following post heat-treatment.

Figure 1 depicts the synthetic process of the CuO nanostructures. First, a large number of Cu(OH)_2_ nuclei were produced by the precipitation reaction between Cu^2+^ and OH^−^ due to the low solubility of Cu(OH)_2_. The resultant Cu(OH)_2_ nuclei clustered together to form some large secondary sheet-like structure during subsequent aging. After heating at 120°C, the Cu(OH)_2_ nanosheets were converted into porous CuO nanosheets following a quasi-topotactic reaction via dehydration. Such structure/phase transition led to formation of rich structural defects due to the incomplete crystallization at low temperature. Next, an additional annealing step was taken to further optimize the crystallinity of the CuO product. The samples prepared by solution route (heated at 120°C), solution reaction followed by annealing at 300, 400, and 500°C were denoted as CuO-120, CuO-300, CuO-400, and CuO-500, respectively for clarity. The relationship of structural aspects and electrochemcial property of the CuO samples were also established.

**Figure 2:**
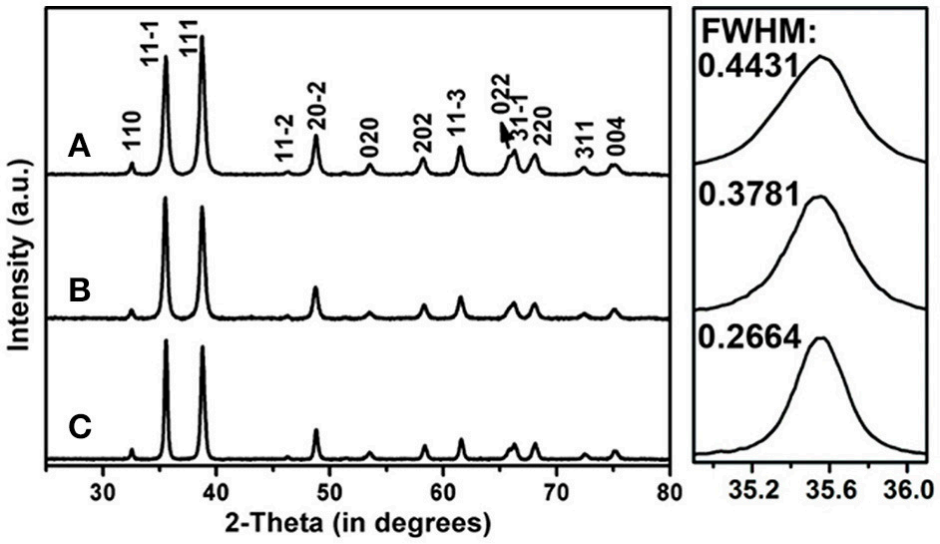
XRD patterns of the CuO nanosheets samples prepared at different conditions. **(A)** CuO-120, **(B)** CuO-300, and **(C)** CuO-500. The right panels show the corresponding enlarged XRD patterns of the selected 2θ regions for better clarity on the FWHM values.

The crystal structures of the CuO products were firstly studied by XRD as shown in Figure 2. All the diffraction peaks of the three samples can be readily indexed to monoclinic CuO (JCPDS card No. 48-1548) (Li et al., 2010; Liu et al., 2012; Zhu et al., 2017). The lack of diffraction peaks for other Cu-based compounds hints the high purity of the as-synthesized CuO products. In addition, the full-width at half-maximum (FWHM) values of the XRD reflections become narrower along with annealing at high temperatures, suggesting the enhanced crystallinity with increased crystallite sizes. As shown in the right panels of Figure 2, the average crystallite sizes of the CuO-120, CuO-300, and CuO-500 samples can be respectively estimated to be *ca*. 18, 22, and 31 nm by Scherrer equation based on the FWHM values of (11-1) planes.

**Figure 3:**
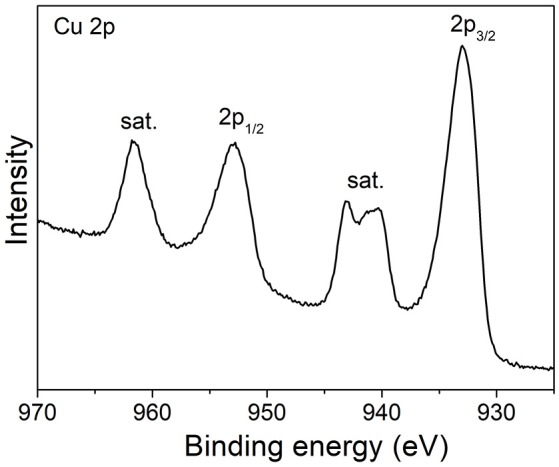
High-resolution Cu 2p XPS spectrum of CuO-300 product.

The surface electronic states of the samples were studied by XPS measurements. Figure 3 shows the high-resolution Cu 2p XPS spectrum of CuO-300 product. The two bands at 933 eV (2p_3/2_) and 952.9 eV (2p_1/2_) along with shake-up satellite peaks at higher binding energies reveal the d^9^ electronic state of Cu^2+^ ions in CuO.

**Figure 4:**
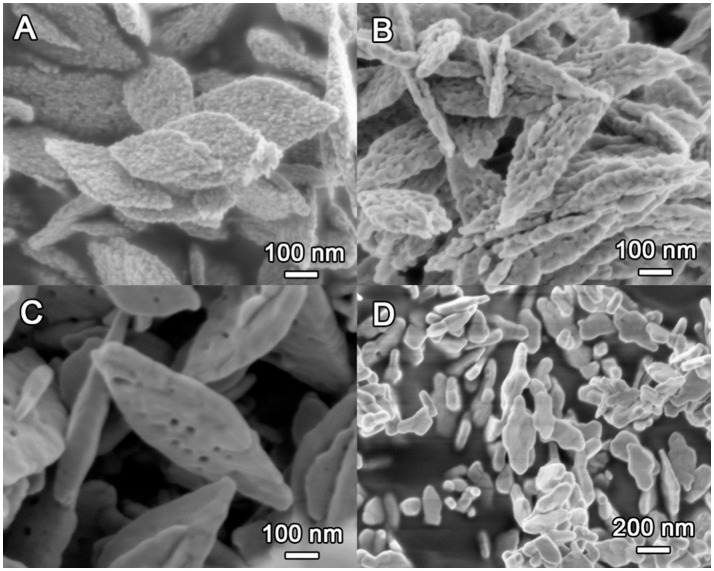
SEM images of the CuO samples prepared at different conditions. **(A)** CuO-120, **(B)** CuO-300, **(C)** CuO-400, and **(D)** CuO-500.

The morphology of the CuO samples was first observed with SEM as depicted in Figure 4. Clearly, the CuO-120 product is composed of many monodisperse, leaf-like nanosheets with lateral sizes of 300–500 nm and thickness of 40–50 nm. These nanosheets are constructed by plenty of interconnected nanoparticles, exhibiting a hierarchical structure with very rough surface (Figure 4A). After annealing at 300°C, the CuO-300 sample (Figure 4B) retains the sheet-like morphology, albeit with the formation of some mesopores on the surface. Note that such CuO nanosheets can be obtained in large quantities as shown in Figure S1. Further increasing annealing temperature to 400°C apparently reduces the number density of the mesopores on the nanosheets of the CuO-400 product (Figure 4C). Meantime, the surface of the CuO nanosheets becomes smooth possibly due to the partial fusion of adjacent nanoparticles. In contrast, the CuO-500 sample (Figure 4D) obtained after annealing at 500°C contains irregular particles without pores. These results suggest that upon annealing at high temperatures the primary nanoparticles can locally migrate or change their position, leading to gradual fusion of adjacent nanoparticles with reduced accessible surface and total surface energy of the whole system. High annealing temperature (e.g., ≥500°C) also tends to destroy the original 2D sheet-like morphology together with severe loss of pore structure.

**Figure 5:**
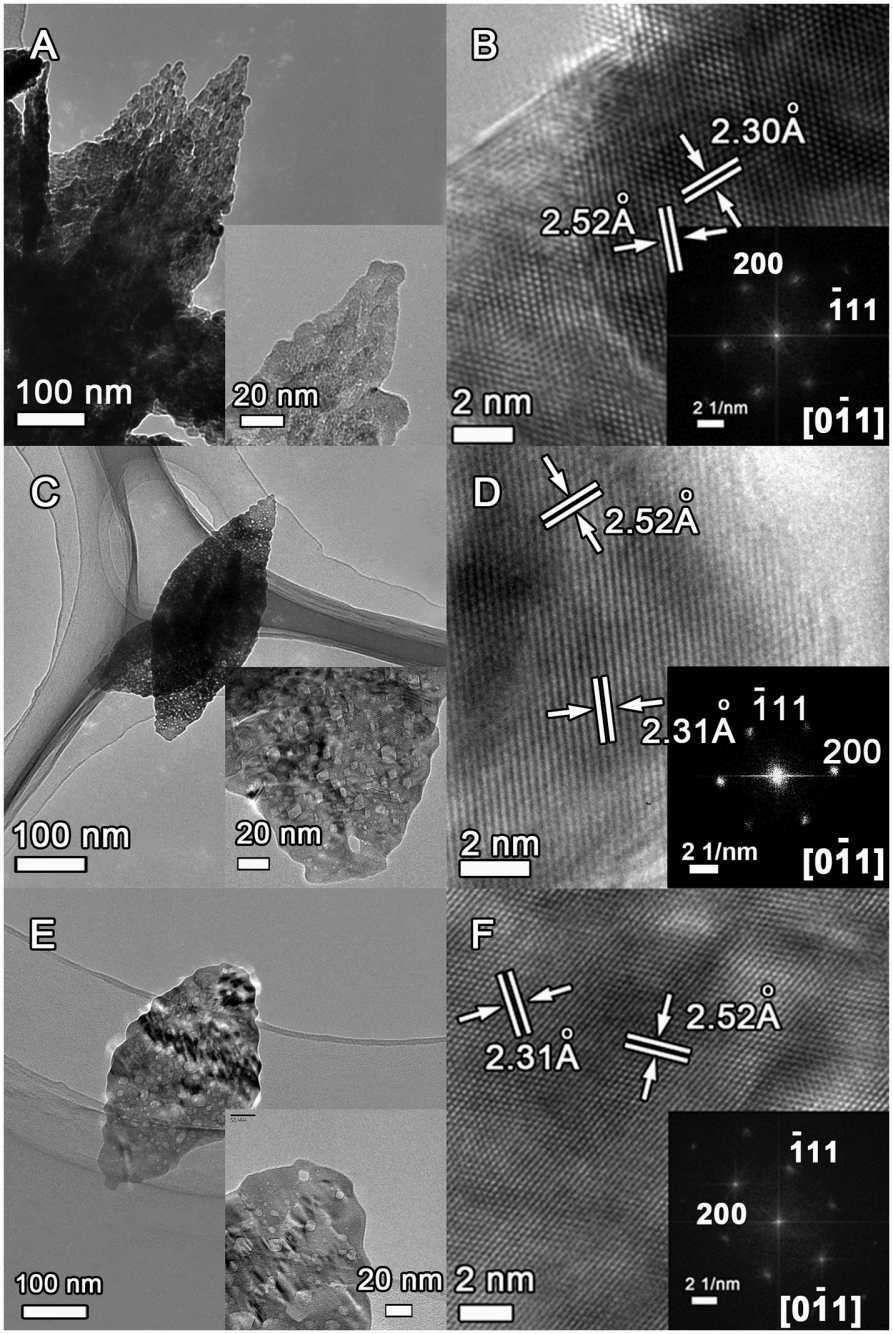
TEM and HRTEM images of the CuO samples prepared under different conditions. **(A,B)** CuO-120, **(C,D)** CuO-300, and **(E,F)** CuO-400.

The microstructure of the as-obtained CuO samples was further investigated by TEM and HRTEM characterizations as shown in Figure 5. The porous leaf-like nanosheets morphology can be clearly observed for the CuO-120, CuO-300, and CuO-400 samples, albeit with the significantly decreased pores in the samples from heat-treatment with increased annealing temperatures (Figures 5A,C,E and their insets). In addition, the clear lattice images in Figures 5B,D,F depict the nanosheets in the three samples are all crystallized. The lattice fringes with interplanar spacing of ~2.31 and ~2.52 Å can be readily indexed to the (200) and (−111) planes of monoclinic CuO phase. Moreover, the corresponding fast Fourier transform (FFT) patterns in the insets of Figures 5B,D,F confirms the single-crystalline nature of the as-obtained CuO nanosheets. However, careful inspection discloses that the diffraction spots in inset of Figure 5B has a slightly weak brightness than that of inset in Figure 5D, suggesting a difference in crystallinity. Nonetheless, the diffraction spots in inset of Figure 5D show somewhat deformation, indicating the possible presence of lattice distortion. In-depth analyses were next performed to probe more details of the possible effects of annealing temperatures on the crystallinity and structural defects in the CuO products. Figure S2 shows the inverse FFT images of the (−111) planes in CuO-120, CuO-300, and CuO-400 samples. Clearly, the CuO-120 product contains plentiful structural distortions (marked by dotted circles) and dislocations (marked by “T”). In CuO-300, the number of these structural defects is obviously reduced. Meantime, the pores are retained and most of them are located on the surface as revealed by HRTEM (Figure S3). Similarly, the number of structural defects further decreases in CuO-400. Therefore, it is anticipated that the electrical transport of the CuO product can be significantly boosted by simply annealing process through removing surface groups and eliminating structural disorders.

**Figure 6:**
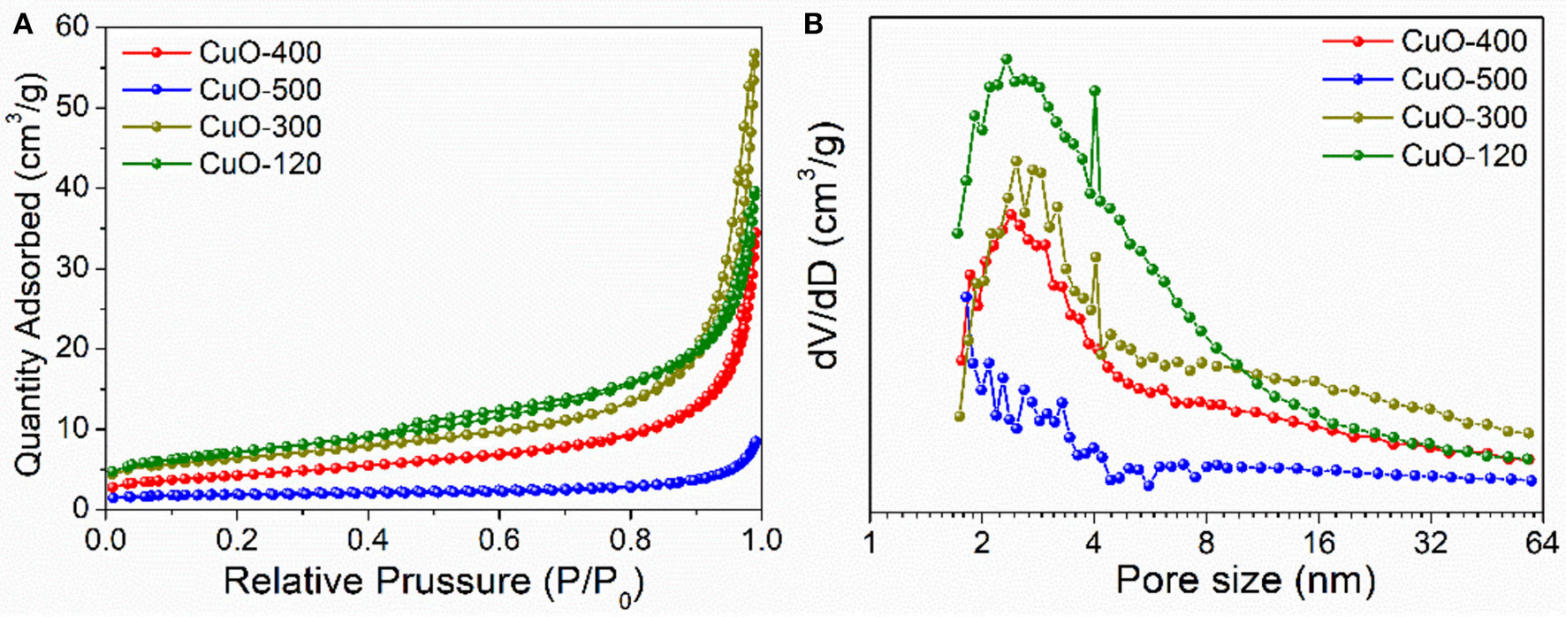
**(A)** N_2_ adsorption–desorption isotherms and **(B)** Pore size distribution plots of CuO nanosheets samples prepared under different conditions.

However, the annealing step may also lead to a considerable change of the surface area of the resultant CuO product which is closely related with the pore structure. For this purpose, N_2_ adsorption experiments were performed in the following. From Figure 5A, the isotherms of the CuO samples display type-III type curves, indicating the presence of slit-like mesopores formed by interparticle stacking. Based on Brunauer-Emmett-Teller (BET) method, the specific surface areas are calculated to be 24.8, 21.8, 15, and 6.1 m^2^/g for CuO-120, CuO-300, CuO-400, and CuO-500 samples, respectively. Clearly, the CuO-300 has a comparable specific surface area to that of CuO-120 due to the well-preserved pore structure. In contrast, the surface area of CuO-500 has been sharply reduced due to the loss of pore and sintering (fusion) of nanoparticle building-blocks. In addition, the pore size distribution of the CuO nanosheets by the Barrett-Joyner-Halenda (BJH) method suggests that all the four samples have a narrow pore size distribution ranging from 1.8 to 4 nm with a maximum pore diameter of ~2.4 nm (Figure 6B), which is roughly in accordance with the SEM and TEM observation. Obviously, the mild synthetic approach and the resultant unique nanosheet morphology of the CuO products contributed to the relatively high specific surface area and pore volume, holding great promise for a series of energy-related applications such as LIBs.

**Figure 7:**
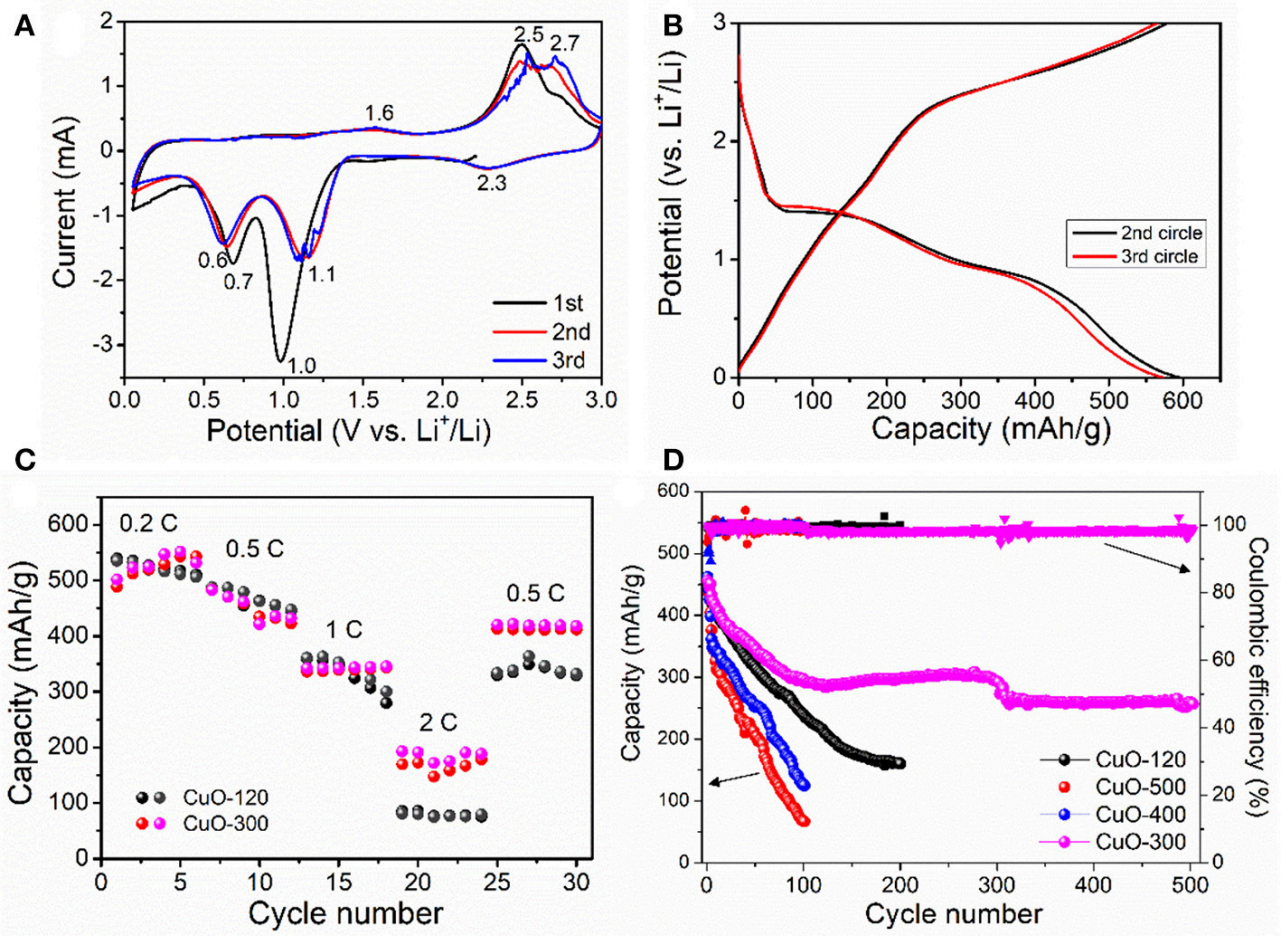
Electrochemical property of the CuO samples. **(A)** Galvanostatic charge/discharge curves, **(B)** Rate property, **(C)** Cyclic voltammetry curves, and **(D)** Cycling performance.

The electrochemical performances of the as-prepared CuO samples were evaluated in CR2025 Li-half cells. Figure 7A depicts the cyclic voltammetry (CV) curves of the CuO-400 electrode within the potential range of 0.01~3 V at a scan rate of 0.2 mV s^−1^. During the first cathodic sweep, a large current response at 1.0 V is noted, which can be mainly due to the formation of solid-electrolyte interface (SEI) layer. In the following cycles, there are three cathodic peaks located at 2.3, 1.1, and 0.6 V, respectively during discharge. The first broad peak at 2.3 V corresponds to the formation of ${\text{Cu}}_{1-\text{x}}^{\text{II}}$${\text{Cu}}_{\text{x}}^{\text{I}}$O_1−x/2_ (0≤x≤0.4) solid solution (Yuan et al., 2017). The second peak at 1.1 V signals the formation of Cu_2_O phase, while the third cathodic peak at 0.6 V correlates to the decomposition of Cu_2_O into metallic Cu and Li_2_O. In the subsequent charge process, three anodic peaks are noted at 1.6, 2.5, and 2.7 V, respectively, which suggests a multistep electrochemical reaction involving the decomposition of SEI film, re-oxidation of Cu to Cu_2_O, and finally to CuO. In addition, the 2nd and 3rd CV curves roughly overlap, indicating the high electrode reversibility. The first discharge-charge profiles of CuO-300 are shown in Figure S4. The CuO-300 electrode displays an initial discharge and charge capacity of 712 and 607 mAh/g, respectively with a Coulombic efficiency of 85%. Figure 7B presents the second and third galvanostatic charge-discharge (GCD) curves of the CuO-300 electrode at 0.2°C. Similarly, three potential plateaus can be noted during the discharging and recharging process, which agrees with that of the CV sweep result. The CuO-300 electrode exhibits a high discharge capacity of 592 mAh/g and charge capacity of 579 mAh/g with a high Coulombic efficiency (CE) of 97.8% in the 2nd cycle, which improves to 99% in the 3rd cycle. Figure 7C compares the rate capability of CuO-120 and CuO-300 electrodes at varied current rates of 0.2–2°C. Both two electrodes demonstrate high and comparable Li-ion storage capacities of ~527, 458, and 337 mAh/g at 0.2, 0.5, 1°C, respectively. However, CuO-300 electrode delivers a higher capacity of 175 mAh/g, higher than that of CuO-120 electrode (80 mAh/g) at 2 C. Note that the rate performance of the CuO-300 is also better than the CuO nanosheets coupled with CNTs (252 mAh/g at 0.75 C, 165.8 mAh/g at 1.5 C) (Yuan et al., 2018). The better rate capacity of CuO-300 electrode at higher current rate can be mainly ascribed to its enhanced electronic conductivity with improved crystallinity and reduced defects. The cycling performance of the four electrodes at 0.5°C is shown in Figure 7D. Clearly, CuO-300 electrode manifests the highest capacity and best cyclability among all the four electrodes. It delivers an initial capacity of 450 mAh/g, which retains 259 mAh/g over 500 cycles with a capacity retention of 58%. This result indicates that the increase of crystallinity is an effective way to improve the cycle stability. In addition, pore structure plays another key role in affecting the Li-storage capability. Note that the cycling properties of CuO-400 and CuO-500 electrodes are poor, even worse than that of CuO-120 electrode. This can be mainly attributed to the sharp reduction of pore structures and specific surface area of the CuO samples subject to heat-treatment at higher temperatures of 400 and 500°C as revealed by the BET results (Figure 6). The electrochemical performance of the CuO-300 sample is also superior to some reported nanostructures, such as pillow-shaped porous CuO with ~320 mAh/g after 50 cycles at 0.1 C (Wan et al., 2011).

**Figure 8:**
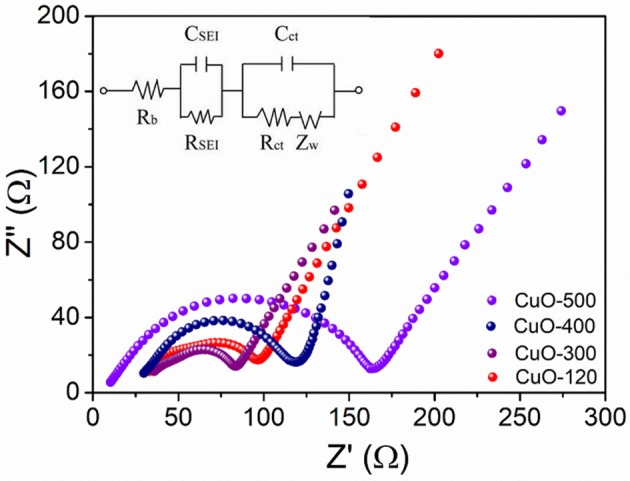
EIS spectra of CuO-120, CuO-300, CuO-400, and CuO-500 electrodes after activation for 5 cycles at 0.2°C.

Electrochemical impedance spectroscopy (EIS) measurement was further performed to probe the kinetic process of the electrode reaction. Figure 8 shows the Nyquist plots of the four electrodes after five cycles at 0.2°C. All the EIS spectra contain two semicircles and a sloping line. The two semicircles at high and high-to-medium frequencies represent the resistances of the SEI film (R_SEI_) and charge transfer (R_ct_) at electrode/electrolyte interface, while the sloping line at low frequency domain depicts the Li^+^ diffusion in the solid state, respectively. Evidently, the CuO-300 electrode shows the lowest R_ct_ value reflected by the smallest diameter of the second semicircle among all the four electrodes, suggesting the lowest polarization and fastest reaction kinetics of the CuO-300 electrode.

## Conclusion

Highly porous single-crystalline CuO nanosheets have been successfully fabricated on a large scale by a facile solution approach followed by heat-treatment at different temperatures. The interplay between electrochemical performance and the structural characteristics (crystallinity, crystallite size, and pore architecture) of the CuO materials has been established. The results indicate that the CuO-300 material delivers the best electrochemical properties in terms of superior rate and stable cycling performance. The superior electrochemical property of the CuO material can be mainly due to the improved crystallinity with boosted electronic conductivity and retained porous structure with highly accessible surface and reduced Li^+^ and electron diffusion path lengths. Our work based on synergetic engineering of structural defects and pore structures may pave the way for development of high-performance electrode materials for next-generation lithium-ion batteries.

## Author contributions

ZD and H-EW conceived and designed the experiments. ZD, ZM, and YaL carried out the experiments and characterizations. YuL, LC, XY and B-LS contributed to data analysis and scientific discussion.

### Conflict of interest statement

The authors declare that the research was conducted in the absence of any commercial or financial relationships that could be construed as a potential conflict of interest.

## References

[B1] Cai Y. Huang S. Z. She F. S. Liu J. Zhang R. L. Huang Z. H. (2016). Facile synthesis of well-shaped spinel LiNi_0.5_Mn_1.5_O_4_ nanoparticles as cathode materials for lithium ion batteries. RSC Adv. 6, 2785–2792. 10.1039/c5ra21723g

[B2] Chai J. Liu Z. Ma J. Wang J. Liu X. Liu H.. (2017). *In situ* generation of poly (vinylene carbonate) based solid electrolyte with interfacial stability for LiCoO_2_ lithium batteries. Adv. Sci. 4:1600377. 10.1002/advs.201600377 28251055 PMC5323859

[B3] Chen J. X. Zhao D. L. Yao R. R. Li C. Wang X. J. Sun F. F. (2017). Hedgehog-like CuO/nitrogen-doped graphene nanocomposite for high-performance lithium-ion battery anodes. J. Alloys Compd. 714, 419–424. 10.1016/j.jallcom.2017.04.171

[B4] Cui X. Song B. Cheng S. Xie Y. Shao Y. Sun Y. (2018). Synthesis of carbon nanotube (CNT)-entangled CuO nanotube networks via CNT-catalytic growth and *in situ* thermal oxidation as additive-free anodes for lithium ion batteries. Nanotechnology 29:11. 10.1088/1361-6528/aa9a23 29130897

[B5] Hu X. S. Li C. Lou X. B. Yang Q. Hu B. W. (2017). Hierarchical CuO octahedra inherited from copper metal-organic frameworks: high-rate and high-capacity lithium-ion storage materials stimulated by pseudocapacitance. J. Mater. Chem. A 5, 12828–12837. 10.1039/c7ta02953e

[B6] Li D. Yan D. Zhang X. Li J. Lu T. Pan L. (2017). Porous CuO/reduced graphene oxide composites synthesized from metal-organic frameworks as anodes for high-performance sodium-ion batteries. J. Colloid Interface Sci. 497, 350–358. 10.1016/j.jcis.2017.03.037 28301830

[B7] Li X. Guo G. Qin N. Deng Z. Lu Z. Shen D. (2018). SnS_2_/TiO_2_ nanohybrids chemically bonded on nitrogen-doped graphene for lithium-sulfur batteries: synergy of vacancy defects and heterostructure. Nanoscale 10, 15505–15512. 10.1039/C8NR04661A 30090890

[B8] Li Y. Yang X. Y. Rooke J. van Tendeloo G. Su B. L. (2010). Ultralong Cu(OH)_2_ and CuO nanowire bundles: PEG200-directed crystal growth for enhanced photocatalytic performance. J. Colloid Interface Sci. 348, 303–312. 10.1016/j.jcis.2010.04.052 20546764

[B9] Liu J. Jin J. Deng Z. Huang S. Z. Hu Z. Y. Wang L.. (2012). Tailoring CuO nanostructures for enhanced photocatalytic property. J. Colloid Interface Sci. 384, 1–9. 10.1016/j.jcis.2012.06.044 22818959

[B10] Liu Y. Liu Y. Cheng S. H. S. Yu S. C. Nan B. Bian H. D. (2016a). Conformal coating of heterogeneous CoO/Co nanocomposites on carbon nanotubes as efficient bifunctional electrocatalyst for Li-air batteries. Electrochim. Acta 219, 560–567. 10.1016/j.electacta.2016.10.064

[B11] Liu Y. Liu Y. Shi H. H. Wang M. Cheng S. H. S. Bian H. D. (2016b). Cobalt-copper layered double hydroxide nanosheets as high performance bifunctional catalysts for rechargeable lithium-air batteries. J. Alloys Compd. 688, 380–387. 10.1016/j.jallcom.2016.07.224

[B12] Lu Z. G. Chen H. L. Robert R. Zhu B. Y. X. Deng J. Q. Wu L. J. (2011). Citric acid- and ammonium-mediated morphological transformations of olivine LiFePO_4_ particles. Chem. Mater. 23, 2848–2859. 10.1021/cm200205n

[B13] Lyu F. Yu S. Li M. Wang Z. Nan B. Wu S.. (2017). Supramolecular hydrogel directed self-assembly of C- and N-doped hollow CuO as high-performance anode materials for Li-ion batteries. Chem. Commun. 53, 2138–2141. 10.1039/c6cc09702b 28134387

[B14] Masse R. C. Liu C. F. Li Y. W. Mai L. Q. Cao G. Z. (2017). Energy storage through intercalation reactions: electrodes for rechargeable batteries. Nat. Sci. Rev. 4, 26–53. 10.1093/nsr/nww093

[B15] Peng H. J. Hao G. X. Chu Z. H. He C. L. Lin X. M. Cai Y. P. (2017). Mesoporous spindle-like hollow CuO/C fabricated from a Cu-based metal-organic framework as anodes for high-performance lithium storage. J. Alloys Compd. 727, 1020–1026. 10.1016/j.jallcom.2017.08.231

[B16] Sun Y. Zhang P. G. Wang B. Wu J. Ning S. S. Xie A. J. (2018). Hollow porous CuO/C nanorods as a high-performance anode for lithium ion batteries. J. Alloys Compd. 750, 77–84. 10.1016/j.jallcom.2018.03.399

[B17] Tan Y. B. Jia Z. Q. Sun J. Y. Wang Y. Z. Cui Z. H. Guo X. X. (2017). Controllable synthesis of hollow copper oxide encapsulated into N-doped carbon nanosheets as high-stability anodes for lithium-ion batteries. J. Mater. Chem. A 5, 24139–24144. 10.1039/c7ta08236c

[B18] Wan M. Jin D. L. Feng R. Si L. M. Gao M. X. Yue L. H. (2011). Pillow-shaped porous CuO as anode material for lithium-ion batteries. Inorg. Chem. Commun. 14, 38–41. 10.1016/j.inoche.2010.09.025

[B19] Wang H. E. Zhao X. Li X. C. Wang Z. Y. Liu C. F. Lu Z. G. (2017). rGO/SnS_2_/TiO_2_ heterostructured composite with dual-confinement for enhanced lithium-ion storage. J. Mater. Chem. A 5, 25056–25063. 10.1039/c7ta08616d

[B20] Wang Z. Zhang Y. Xiong H. Qin C. Zhao W. Liu X. (2018). Yucca fern shaped CuO nanowires on Cu foam for remitting capacity fading of Li-ion battery anodes. Sci. Rep. 8:10. 10.1038/s41598-018-24963-2 29695815 PMC5916934

[B21] Wu S. Wang W. Li M. Cao L. Lyu F. Yang M.. (2016). Highly durable organic electrode for sodium-ion batteries via a stabilized alpha-C radical intermediate. Nat. Commun. 7:13318. 10.1038/ncomms13318 27819293 PMC5103065

[B22] Wu S. Zhu Y. Huo Y. Luo Y. Zhang L. Wan Y. (2017). Bimetallic organic frameworks derived CuNi/carbon nanocomposites as efficient electrocatalysts for oxygen reduction reaction. Sci. China Mater. 60, 654–663. 10.1007/s40843-017-9041-0

[B23] Xi L. J. Wang H. E. Lu Z. G. Yang S. L. Ma R. G. Deng J. Q. (2012). Facile synthesis of porous LiMn_2_O_4_ spheres as positive electrode for high-power lithium ion batteries. J. Power Sources 198, 251–257. 10.1016/j.jpowsour.2011.09.100

[B24] Yin D. M. Huang G. Na Z. L. Wang X. X. Li Q. Wang L. M. (2017). CuO nanorod arrays formed directly on Cu foil from MOFs as superior binder-free anode material for lithium-ion batteries. ACS Energy Lett. 2, 1564–1570. 10.1021/acsenergylett.7b00215

[B25] Yin H. Yu X. X. Li Q. W. Cao M. L. Zhang W. Zhao H. (2017). Hollow porous CuO/C composite microcubes derived from metal-organic framework templates for highly reversible lithium-ion batteries. J. Alloys Compd. 706, 97–102. 10.1016/j.jallcom.2017.02.215

[B26] Yuan W. Luo J. Pan B. Y. Qiu Z. Q. Huang S. M. Tang Y. (2017). Hierarchical shell/core CuO nanowire/carbon fiber composites as binder- free anodes for lithium- ion batteries. Electrochim. Acta 241, 261–271. 10.1016/j.electacta.2017.04.159

[B27] Yuan W. Qiu Z. Chen Y. Zhao B. Liu M. Tang Y. (2018). A binder-free composite anode composed of CuO nanosheets and multi-wall carbon nanotubes for high-performance lithium-ion batteries. Electrochim. Acta 267, 150–160. 10.1016/j.electacta.2018.02.081

[B28] Zhang J. Cui Y. X. Qin Q. Zhang G. F. Luo W. H. Zheng W. J. (2018). Nanoporous CuO mesocrystals: low-temperature synthesis and improved structure-performance relationship for energy storage system. Chem. Eng. J. 331, 326–334. 10.1016/j.cej.2017.08.089

[B29] Zhang Q. B. Chen H. X. Luo L. L. Zhao B. T. Luo H. Han X. (2018). Harnessing the concurrent reaction dynamics in active Si and Ge to achieve high performance lithium-ion batteries. Energy Environ. Sci. 11, 669–681. 10.1039/c8ee00239h

[B30] Zhang Q. B. Zhang K. L. Xu D. G. Yang G. C. Huang H. Nie F. D. (2014). CuO nanostructures: synthesis, characterization, growth mechanisms, fundamental properties, and applications. Prog. Mater Sci. 60, 208–337. 10.1016/j.pmatsci.2013.09.003

[B31] Zhao C. Cai Y. Yin K. Li H. Shen D. Qin N. (2018). Carbon-bonded, oxygen-deficient TiO_2_ nanotubes with hybridized phases for superior Na-ion storage. Chem. Eng. J. 350, 201–208. 10.1016/j.cej.2018.05.194

[B32] Zheng Z. Zao Y. Zhang Q. Cheng Y. Chen H. Zhang K. (2018). Robust erythrocyte-like Fe_2_O_3_@carbon with yolk-shell structures as high-performance anode for lithium ion batteries. Chem. Eng. J. 347, 563–573. 10.1016/j.cej.2018.04.119

[B33] Zhou H. Kang M. Qin B. Zhao N. Wu D. Lv B. L. (2018). Glucose-mediated template-free synthesis of hollow CuO microspheres. RSC Adv. 8, 14157–14163. 10.1039/c8ra00684a PMC9079924 35540754

[B34] Zhu J. Shang C. Q. Wang Z. Y. Zhang J. J. Liu Y. Gu S. (2018). SnS/SnSb@C nanofibers with enhanced cycling stability via vulcanization as an anode for sodium-ion batteries. Chemelectrochem 5, 1098–1104. 10.1002/celc.201701270

[B35] Zhu Q. Wang M. Nan B. Shi H. H. Zhang X. M. Deng Y. H. (2017). Core/shell nanostructured Na_3_V_2_(PO_4_)_3_/C/TiO_2_ composite nanofibers as a stable anode for sodium-ion batteries. J. Power Sources 362, 147–159. 10.1016/j.jpowsour.2017.07.004

