# Supplementary material for: Boosting Lithium-Ion Storage Capability in CuO Nanosheets via Synergistic Engineering of Defects and Pores
Source: Front Chem. 2018 Sep 24;6:428. doi: 10.3389/fchem.2018.00428 (PMC6166579; doi:10.3389/fchem.2018.00428)
Supplement: Supplementary file 1 [file Data_Sheet_1.docx]

**Supporting Information**


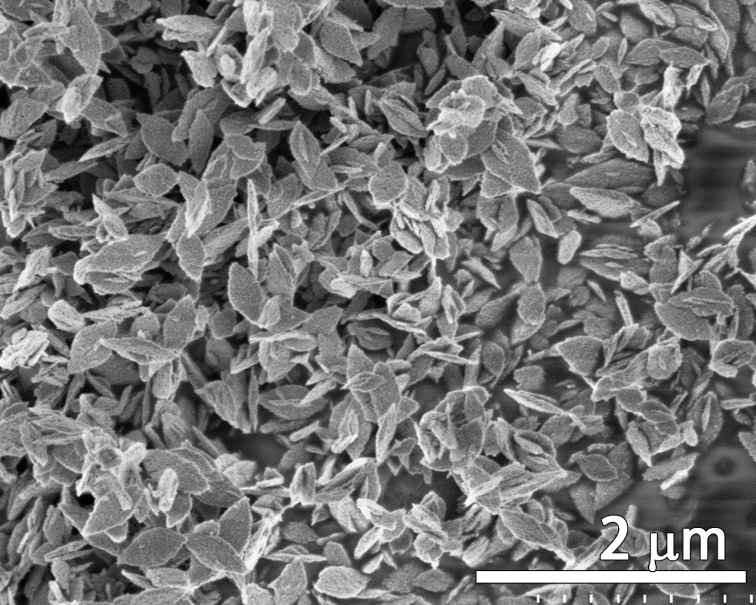


**Figure S1.** Low-magnification SEM image of CuO-300 sample.


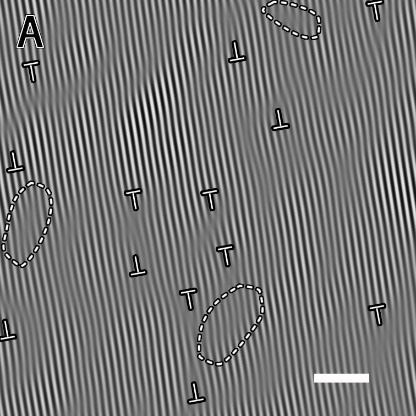

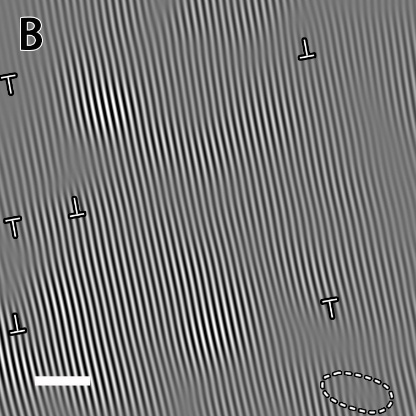

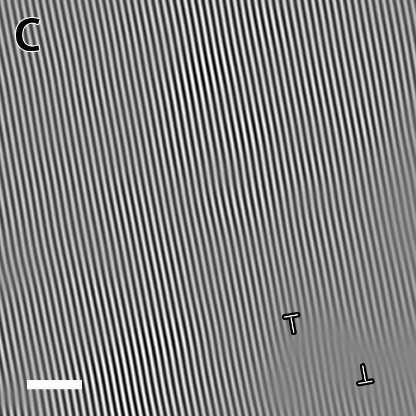


**Figure S2.** FFT patterns of the CuO samples images shows the edge dislocation at the (-111) plane (A) CuO-120, (B) CuO-300 and (C) CuO-400. Dislocations are indicated by white “T”. Dashed lines in (A) and (B) indicate not only edge dislocations exist. (Scale bar: 5nm).


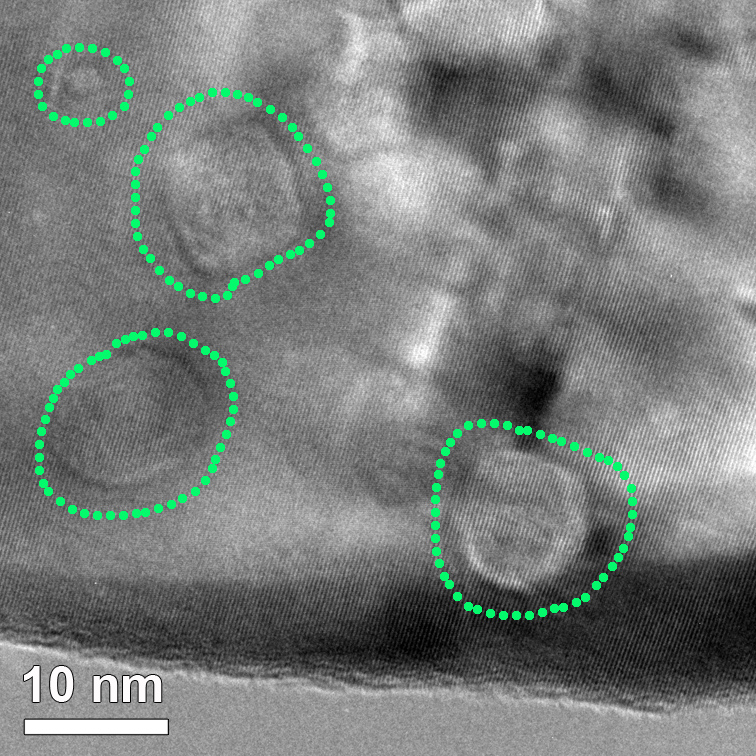


**Figure S3.** HRTEM image of the pore structure of the CuO-300 sample.





**Figure S4**. First discharge-charge curves of the CuO-300 electrode at 0.2 C.
